# An unusual hantavirus outbreak in southern Argentina: person-to-person transmission? Hantavirus Pulmonary Syndrome Study Group for Patagonia.

**DOI:** 10.3201/eid0302.970210

**Published:** 1997

**Authors:** R M Wells, S Sosa Estani, Z E Yadon, D Enria, P Padula, N Pini, J N Mills, C J Peters, E L Segura

**Affiliations:** Centers For Disease Control and Prevention, Atlanta, Georgia, USA.

## Abstract

Hantavirus pulmonary syndrome is a rodent-borne zoonosis first recognized in the United States in 1993. Person-to-person transmission has not been reported; however, in the outbreak of 20 cases reported here, epidemiologic evidence strongly suggests this route of transmission.


### An Unusual Hantavirus Outbreak in Southern Argentina: Person-to-Person Transmission?

Hantavirus pulmonary syndrome is a rodent-borne zoonosis first recognized in the United States in 1993. Person-to-person transmission has not been reported; however, in the outbreak of 20 cases reported here, epidemiologic evidence strongly suggests this route of transmission.

In 1995, a novel hantavirus (Andes virus) was identified in samples from patients in southern Argentina (1). The patients had hantavirus pulmonary syndrome (HPS), a disease first described 2 years earlier in the United States, in association with Sin Nombre virus (a New World hantavirus) (2,3). Old World hantaviruses (Puumala, Seoul, Hantaan, and Dobrava) have been recognized causes of hemorrhagic fever with renal syndrome (HFRS) for at least 40 years and are responsible for more than 100,000 cases each year in Eurasia (4). Hantaviruses (family Bunyaviridae, genus *Hantavirus*) are rodent-borne viral zoonotic agents believed to be transmitted to humans primarily by inhalation of aerosols of infected rodent excretions (4). Person-to-person transmission of hantaviruses has not been reported despite extensive epidemiologic studies. Between September 22 and December 5, 1996, 18 cases of HPS occurred in residents of, or visitors to, the towns of El Bolsón, Bariloche, and Esquel in southern Argentina. Two additional persons who had contact with El Bolsón patients but had not visited the area, contracted HPS during this period (5). Five of the patients were physicians; three were directly responsible for the clinical care of an HPS patient. Epidemiologic links between all but four of the cases and evidence of low rodent population density in the area strongly suggest person-to-person transmission of HPS during this outbreak.

Sporadic cases of HPS and possible cases of HFRS from as early as 1987, have been retrospectively identified in Argentina (6). In 1995, genetic material from Andes virus, a unique hantavirus, was identified in the lungs of a patient from El Bolsón (population ~15,000) in southern Argentina (1). The outbreak of HPS reported in this dispatch began on September 22, 1996, when the 41-year-old index patient (patient I) (also from El Bolsón) became ill with HPS (Figure 1). El Bolsón, a rural town in Rio Negro Province, is approximately 350 meters above sea level in the foothills of the Andes. Patients requiring intensive care are transferred to Bariloche

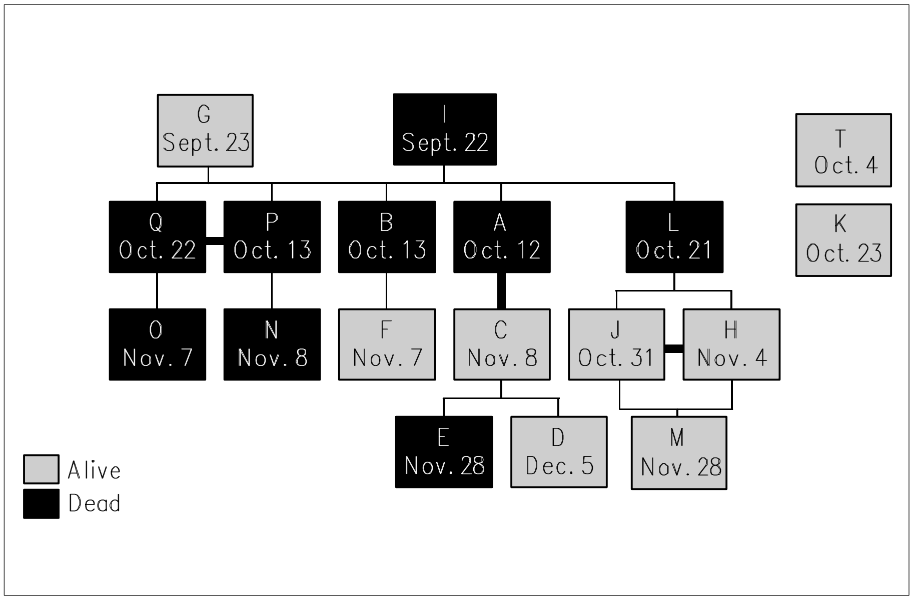

Figure 1. Transmission tree for HPS cases in southern Argentina, September–December 1996, indicating dates of onset of symptoms, survivor status, and proposed lines of transmission. Lines of transmission are hypothetical since many of the patients had contact with multiple HPS patients. Bold lines denote husband and wife. The two sporadic cases, U and R, are not shown.

(population 80,000), 150 kilometers to the north (Figure 2).

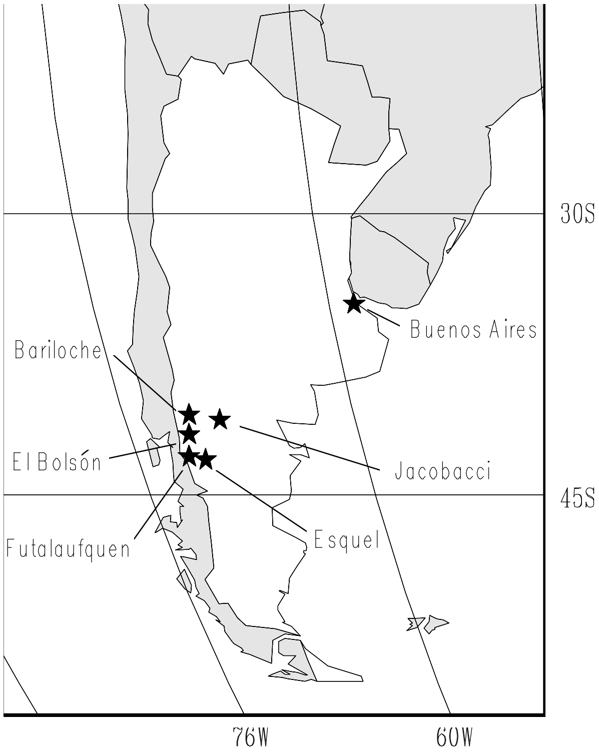

Figure 2. Towns involved in the 1996 HPS outbreak in southern Argentina.

Potential cases were identified by local physicians familiar with the clinical features of HPS (3) and were accepted as cases when enzyme-linked immunosorbent assay detected IgM antibodies reacting with Sin Nombre virus and/or when reverse transcriptase and polymerase chain reaction detected hantaviral RNA (1,2,6,7). Viral sequences indicated a sigmodontine-derived hantavirus related to, but clearly distinct from, Sin Nombre virus; additional sequencing and phylogenetic studies are under way.

Twenty-one and 20 days, respectively, after the index patient became symptomatic, his 70-year-old mother (patient B) and one of his doctors (patient A) contracted HPS. The doctor's spouse, also a doctor (patient C), became ill with HPS 27 days after her husband's first symptoms (19 days after his death). She traveled to Buenos Aires for medical care. In a Buenos Aires hospital, an admitting doctor (patient D) spent 1 hour taking a clinical history and examining her. The doctor (patient D) applied pressure to a venipuncture site on patient C's arm with multiple layers of gauze; no obvious blood contact occurred. The only other contact between this doctor and patient C occurred 2 days later, when the doctor briefly visited the hospital's intensive care unit to attend to another patient. Twenty-four days after attending patient C, the doctor became ill with HPS. The doctor, patient D, had not traveled outside Buenos Aires, and she reported no contact with rodents during the 2 months preceding her illness (5).

A 40-year-old doctor (patient E) from Buenos Aires contracted HPS 17 days after patient C was admitted to the hospital. This doctor was a friend of patients A and C and spent 3 days in El Bolsón after the death of patient A. She visited patient C often in the hospital but was not directly involved in the clinical management of any HPS patients. A fifth doctor with HPS (patient F) reported having close contact with several HPS patients. He intubated patient B and examined patients I and G. He also had daily contact with his colleague, (patient A), and spoke briefly with patient I's sister (patient H), brother-in-law (patient J), and friend (patient K).

At the funeral of patient B, patient I's housekeeper (patient L) was already symptomatic. She returned to Buenos Aires by car with patients H, J, and their daughter (patient M). Symptoms developed in the latter three occupants of the car 11, 15, and 29 days, respectively, after the drive back to Buenos Aires. No evidence of rodent infestation was found when the car was examined 3 weeks later. Although patients H and J had stayed at the home of patient I for several nights, patient M had not been to El Bolsón in the preceding months, traveling only as far as Jacobacci (~250 miles from El Bolsón) for patient B's funeral.

A second group of HPS cases occurred in Bariloche. Four people who had visited or worked in the hospital to which many of the El Bolsón patients were transferred (a modern 40-bed facility with a three-bed intensive care unit) contracted HPS: the hospital's night receptionist (patient N), a 40-year-old woman (patient O) who visited an unrelated patient, a 27-year-old man (patient P), and his wife (patient Q). Patients P and Q developed a close relationship with patient N during their many visits to the hospital between September 20 (when their 27-week-gestation baby was born) and October 27 (when the baby died). The three shared mate (a local tea drunk through a communal metal straw), and patient P occasionally rested on patient N's camp bed. The baby got abdominal distention and shock 37 days after birth. The clinical signs suggested necrotizing enterocolitis, but no serum or tissue specimens from the infant were available for definitive diagnosis.

The remaining three HPS patients in El Bolsón during this period were men aged 44 (patient G), 29 (patient R), and 14 (patient T) years. Patients G and T were friends or acquaintances of one or more of the HPS patients, but did not recall contact with an HPS patient in the 6 weeks before the onset of their symptoms.

Both patients R and U likely had coincidental sporadic cases of HPS. Patient R lived in the Chilean mountains and had prodromal symptoms of HPS on arrival in El Bolsón. Patient U, a 33-year-old man living and working in Futalaufquén National Park, Chubut Province (150 km south of El Bolsón), also contracted HPS during this period but is unlikely to have had contact with HPS patients in El Bolsón.

Overall, 11 (55%) of the 20 patients were male, the mean age was 38 years (range 13-70 years), and the case-fatality rate was 50%. The death rate among cases for which epidemiologic links were identified (Figure 1) was lower during the latter stage of the outbreak (three of nine patients with onset of symptoms in November-December died compared with six of seven patients with onset of symptoms in October). The clinical picture was similar to that of HPS in the United States, although several atypical features, such as conjunctival injection and head and neck suffusion, were noted. Coughing did not occur earlier or more frequently in Argentinean HPS patients than in U.S. patients, which suggests that coughing was not the critical factor in person-to-person transmission.

Early in the investigation of this outbreak, attention was focused on the index patient's brick house, one of several dwellings located on a semirural property on the outskirts of El Bolsón. Several weeks before patient I became sick, he and patients B and L moved from a wooden cabin into the brick house. Two doctors (patients A and F) made visits to this house, and patients H and J stayed there at least one night during November 1996. The 13 other HPS patients in the outbreak did not visit the house. One month after the onset of patient I's symptoms, an examination of the brick house and other nearby structures found the buildings to be well constructed with no obvious sites for rodent entry and no evidence of rodent infestation (as confirmed by trapping studies). Trapping in and around the homesites of the El Bolsón and Bariloche patients yielded few rodents. Trap success (i.e., the number of rodents captured per 100 trap nights) in neighboring areas of disturbed or natural vegetation indicated a low rodent population density. Identification and serologic testing of captured rodents are under way. The low trap success rate contrasted with that of the 1993 HPS outbreak in the southwestern United States, when rodents were plentiful overall and significantly more numerous in and around homes of patients than in and around homes of controls (8).

The probable transmission of hantavirus from patient C to her doctor (Patient D) is the best epidemiologic evidence available to support the hypothesis that person-to-person transmission of hantavirus occurred during this outbreak. When considered separately, links between other Argentinean HPS patients are less convincing. However, when the contacts between patients are viewed collectively, the probability that person-to-person transmission played a major role in this outbreak strengthens. A comparison of the relative success of rodent trapping efforts over the last 20 years indicated that rodent population densities in the general area of the outbreak during the spring of 1996 were lower than average (O. Pearson, pers. comm. 1996), adding further support for the hypothesis of person-to-person transmission. It is highly unlikely that the low rodent population density observed was the result of a precipitous decline in rodent populations in late spring. Rodent populations in northwest Patagonia are at minimal levels in the early spring and represent only animals that have survived the winter. Renewed reproduction increases population density throughout the spring (N. Guthmann, pers. comm. 1997), as was corroborated by the high percentage of immature animals captured during trapping. Thus the rodent population density during our trapping in late spring is likely to have been higher than density when the outbreak began.

The unique pattern of person-to-person transmission in this outbreak has not been a feature of hantavirus epidemiology; in fact, a study performed in New Mexico in 1993 found no disease or hantavirus antibodies in serum specimens taken from 396 health care workers, including 266 who had been exposed to patients with HPS or their body fluids 2 to 6 weeks earlier (9). The high case-fatality ratio has precluded a detailed description of the exact nature of contact between cases. Consequently, the likely mode of transmission—whether through direct contact, droplets, infectious aerosols, or contaminated fomites—is not known. Because hantaviruses are difficult to isolate and cultivate, few attempts have been made to study virus excretion from patients or to assess viral stability under different environmental conditions. Efforts to define the infectious stage of the disease and the type and duration of contact that may have led to transmission are continuing. Genetic sequences of viruses from patients and rodents are being determined to define more clearly the pattern of transmission. In the absence of any clear-cut evidence for nosocomial transmission of Sin Nombre or related viruses in the United States, it is premature to suggest changing the existing guidelines for care of HPS patients. The level of suspicion should be raised to investigate any suspected person-to-person spread of this rodent-borne zoonosis, however. Further epidemiologic and laboratory studies are needed to clarify requirements for protecting healthcare workers and others from hantaviral infections in Patagonia.

**Rachel M. Wells,* Sergio Sosa Estani,† Zaida E. Yadon,‡ Delia Enria,¶ Paula Padula,‡ Noemi Pini,† James N. Mills,* Clarence J. Peters,* Elsa L. Segura,‡ and the Hantavirus Pulmonary Syndrome Study Group for Patagonia§**

*Centers For Disease Control and Prevention, Atlanta, Georgia, USA; †Instituto Nacional de Chagas "Dr. Mario Fatala Chaben", Buenos Aires, Argentina; ‡Administración Nacional de Laboratorios e Institutos de Salud, Buenos Aires, Argentina; ¶Instituto Nacional de Enfermedades Virales Humanas "Dr. Julio I. Maiztegui," Buenos Aires, Argentina

§N. Guthmann, Centro Regional Universitario Bariloche; E. Argüelo, F. Klein, R. Levy, C. Nagel, Fundación Favorolo; R. Calfin, F. de Rosas, M. Lazaro, M. Rosales, P. Sandoval, Hospital Bariloche; L.A. Albornoz, R. L. Antonia, J. C. Becerra, W.H. Benitez, A. Broide, C. Flandes, A. Honik, J. Meza, L. Paganini, A. Resa, G. Roberts, P. Rodriguez, I.E. Rojo, G. Suppo, A. Werenicz, S. Wisky, Hospital El Bolson; H. Quiroga, Hospital El Hoyo; C.A. Cohen Arazi, L. Bogni, R. Lombardelli, B. Salvagno, C. Simeone, Hospital Esquel; L. Clara, Hospital Italiano de la Ciudad de Buenos Aires; M. del Castillo, Hospital Nacional de Clínicas "José de San Martín"; J.L. Becker, H. Lopez, M. Palmigiani, Instituto Nacional de Enfermedades Virales Humanas "Dr. Julio I. Maiztegui"; M. Ameztoy, A. Lawrynowicz, Instituto Nacional de Epidemiología "Dr. Juan H. Lara"; A. Edelstein, Instituto Nacional de Microbiología "Dr. Carlos Malbran"; A. Aguilera, V.O. Vigil, Instituto Nacional de Parasitología "Dr. Mario Fatala Chaben"; R. Chuit, C. Riva Posse, Ministerio de Salud y Acción Social; C. Barclay, L. Caride, E. De Orta, M. Furque, A. Picone, L. Samengo, Sanatorio San Carlos; R. Fernandez, R. Gonzales, Sistema Provincial de Salud, Esquel; O. Arellano, G. Cantoni, E. Larrieu, J. Vilosio, Sistema Provincial de Salud, PCIA de Río Negro; K. Busico, A. Khan, T.G. Ksiazek, W. Terry, J. Young, S. Zaki, Centers for Disease Control and Prevention

### References

1. Lopez N, Padula P, Rossi C, Lazaro ME, Franze-Fernández MT. Genetic identification of a new hantavirus causing severe pulmonary syndrome in Argentina. Virology 1996;220:223-6.
2. Nichol ST, Spiropoulou CF, Morzunov S, Rollin P, Ksiazek TG, Feldmann H, et al. Genetic identification of a hantavirus associated with an outbreak of acute respiratory illness. Science 1993;262:914-7
3. Khan A, Khabbaz RF, Armstrong L, Holman RC, Bauer SP, Graber J, et al. Hantavirus pulmonary syndrome: the first 100 cases. J Infect Dis 1996;173:1297-303
4. McKee KT, LeDuc JW, Peters CJ. Hantaviruses. In: Belshe R, editor. Textbook of human virology, 2nd edition. St. Louis: Mosby Year Book, Inc., 1991:615-32
5. Enria D, Padula P, Segura EL, Pini N, Edelstein A, Riva Posse C, et al. Hantavirus pulmonary syndrome in Argentina. Possibility of person to person transmission. Medicina (B Aires) 1995;58:709-11.
6. Parisi M, Enria D, Pini NC, Sabatini MS. Detección retrospectiva de infecciones clínicas por hantavirus en la Argentina. Medicina (B Aires) 1995;56:1-13.
7. Ksiazek TG, Peters CJ, Rollin PE, Zaki S, Nichol S, Spiropoulou C, et al. Identification of a new North American hantavirus that causes acute pulmonary insufficiency. Am J Trop Med Hyg 1995;52:1017-23.
8. Childs JE, Krebs JW, Ksiazek TG, Maupin GO, Gage KL, Rollin PE , et al. A household-based, case-control study of environmental factors associated with hantavirus pulmonary syndrome in the southwestern United States. Am J Trop Med Hyg 1995;52:393-7.
9. Vitek CR, Breiman RF, Ksiazek TG, Rollin PE, McLaughin JC, Umland ET, et al. Evidence against person-to-person transmission of hantavirus to health care workers. Clin Infect Dis 1996;22:824-6.
